# Wavelet analyses of electromyographic signals derived from lower extremity muscles while walking or running: A systematic review

**DOI:** 10.1371/journal.pone.0206549

**Published:** 2018-11-02

**Authors:** Irene Koenig, Patric Eichelberger, Angela Blasimann, Antonia Hauswirth, Jean-Pierre Baeyens, Lorenz Radlinger

**Affiliations:** 1 Bern University of Applied Sciences, Department of Health Professions, Division of Physiotherapy, Bern, Switzerland; 2 Vrije Universiteit Brussel, Faculty of Physical Education and Physiotherapy, Brussels, Belgium; University of Illinois at Urbana-Champaign, UNITED STATES

## Abstract

Surface electromyography is often used to assess muscle activity and muscle function. A wavelet approach provides information about the intensity of muscle activity and motor unit recruitment strategies at every time point of the gait cycle. The aim was to review papers that employed wavelet analyses to investigate electromyograms of lower extremity muscles during walking and running. Eleven databases were searched up until June 1^st^ 2017. The composition was based on the PICO model and the PRISMA checklist. First author, year, subject characteristics, intervention, outcome measures & variables, results and wavelet specification were extracted. Eighteen studies included the use of wavelets to investigate electromyograms of lower extremity muscles. Three main topics were discussed: 1.) The capability of the method to correctly assign participants to a specific group (recognition rate) varied between 68.4%-100%. 2.) Patients with ankle osteoarthritis or total knee arthroplasty presented a delayed muscle activation in the early stance phase but a prolonged activation in mid stance. 3.) Atrophic muscles did not contain type II muscle fiber components but more energy in their lower frequencies. The simultaneous information of time, frequency and intensity is of high clinical relevance because it offers valuable information about pre-and reflex activation behavior on different walking and running speeds as well as spectral changes towards high or low frequencies at every time point of the gait cycle.

## Introduction

Electromyograms (EMG) is a commonly used method to measure muscle activity and to assess muscle function [1–5]. While running and walking, one will find periodic changes in muscle activity as joints undergo acceleration and deceleration throughout the gait cycle. Conventional analyses of EMG signals provide information about the intensity of muscle activity over time [1]. Unfortunately, there is no information about the frequency content. The frequency is needed to make an estimation about the activated alpha-motoneurons and related fiber types. The small alpha-motoneurons and related slow type I fibers are responsible for the lower frequencies in the signal and the large alpha-motoneurons and their related fast type II fibers for the higher frequencies [6]. To analyse the frequency components, Fourier Transform (FT) is often used [2,3]. The FT method provides a frequency spectrum from which the mean or median frequency can be extracted to quantify the frequency content of a signal. Nevertheless, no information can be retrieved from FT about the time instances when these frequencies occur. Therefore, an important precondition for the application of a FT analysis is that the frequency of a signal must not change over time (stationarity). However, this is not the case for dynamic muscle activity, hence the FT approach is basically not applicable to analyze whole body movements [4,5]. Wavelet approaches provide a solution to analyze time-, frequency- and intensity-behavior of the electromyographic signal simultaneously [1]. This advantage allows to evaluate patterns generated muscle activation during different walking and running tasks [7–10], which are characterized by changing frequencies over time (non-stationary nature of a signal). A wavelet approach extracts the frequencies over a period of time. Therefore, the wavelet method distinguishes between activities of slow type I muscle fibers (lower frequencies) and the fast type II fibers (higher frequencies) [7]. Summarized, wavelet analyses of an electromyographic signal allow the extraction of intensities and frequencies with a high time resolution. Because of these benefits, wavelets are often used in gait analysis [7–10]. This systematic review aimed to summarize the application of wavelets to analyze the activity of the lower extremity muscles in different walking, running and orthopedic conditions.

## Methods

### Data sources and searches

This systematic review followed the PRISMA guidelines and the PICO model for the definition of the inclusion criteria: P (Population): “human adults”, I (Intervention): “impact of gait, running or walking on muscle activity of the lower extremities”, C (Control): “healthy or different gait conditions” and O (Outcome): “EMG wavelet parameters” [11]. Neurological diseases were excluded because of the disease-related, specific gait patterns and the possible sensorimotor dysfunction. This systematic review has been listed in the international prospective register of systematic reviews (PROSPERO) with the identification number CRD42016035986.

The electronic databases PubMed/MEDLINE, EMBASE, CINAHL, Cochrane Library, PEDRO, SURF, Art Source, Business Source Premier, Green File and Health Source as well as the search engine Google Scholar were systematically searched with the search terms wavelet AND (EMG OR electromyography) AND (running OR walking OR gait). Additionally, reference lists from retrieved publications were hand-searched. The languages were restricted to English, German, French, Italian, Spanish and Dutch. All study designs, except systematic reviews, were included. The search was completed on June 1^st^ 2017.

### Data extraction and quality assessment

Two investigators experienced in signal analysis performed the whole data extraction process independently. Discussion took place after every fulfilled part of the protocol until consensus was achieved. The investigators independently screened all titles for eligibility, based on the a priori defined PICO inclusion criteria. In case of disagreement, consensus was achieved through discussion. In case of uncertainty, the study was taken to the next step. Afterwards, the same process was performed to screen the abstracts and finally to screen the full texts for eligibility. If no consensus was found after the discussion, a third investigator decided whether the study should be included or excluded. In the further process, the quality of the included studies and the risk of bias were analyzed using “The Cochrane Collaboration`s tool for assessing risk of bias”. The criteria list included six items from which each was scored with “+” if the criteria was fulfilled, with “-”if the criteria was not fulfilled or with “?” if the data was not provided or unclear. According to Viswanathan (2012) [12] the downgrade of the studies not designed as a RCT was limited by rating the selection bias as unclear risk of bias if the method was appropriate.

### Data synthesis and analysis

Next, the following data were independently extracted and analyzed by two investigators using a standardized extraction form: First author, year; subject characteristics; intervention; outcome: measures & variables; results; wavelet specification.

## Results

**Fig 1:**
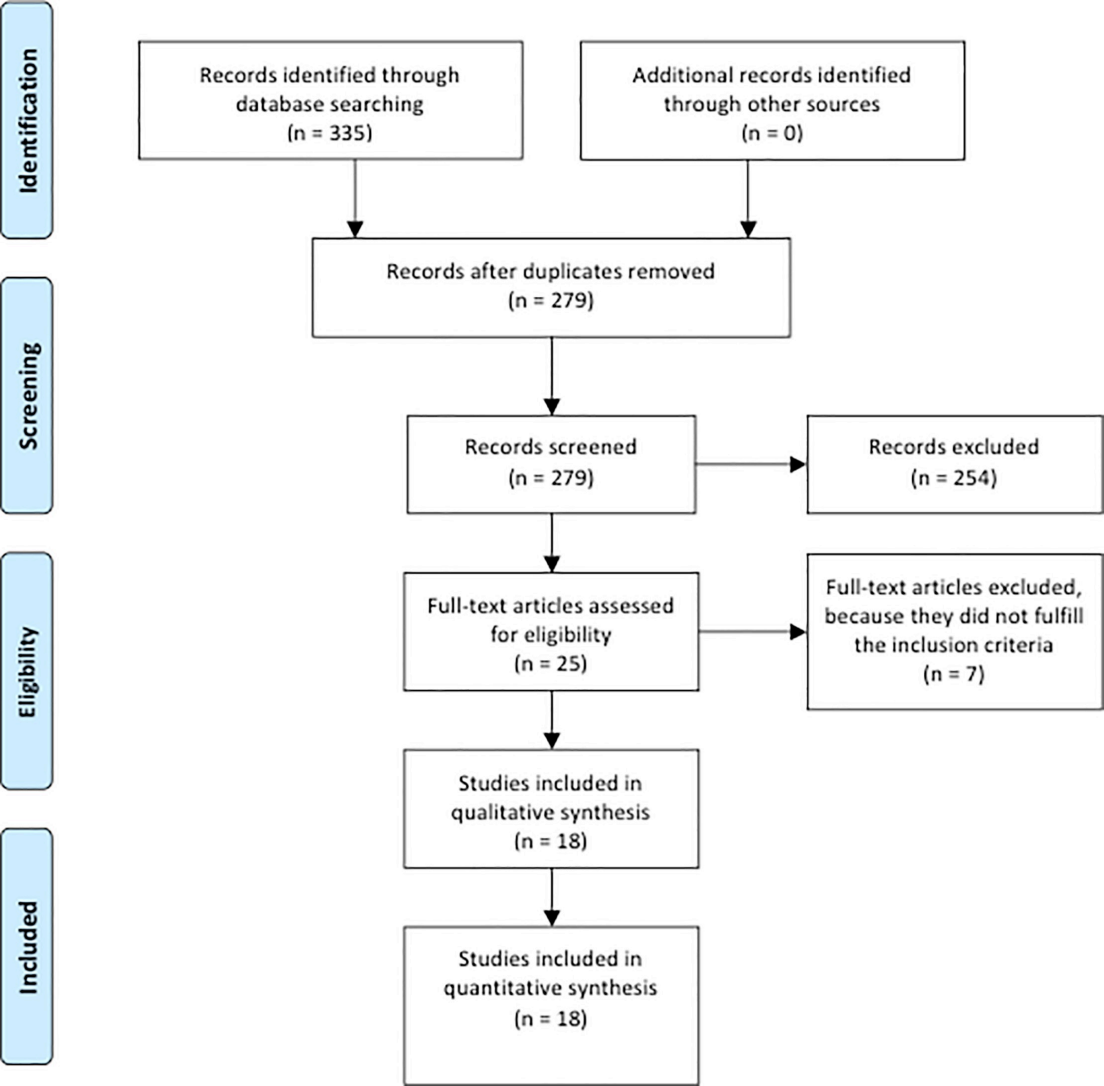
PRISMA 2009 flow diagram.

Fig 1 presents the flow chart for the selection of studies. The literature search revealed a total of 335 records. After removal of duplicates, 279 papers remained to be screened on titles and abstracts. A number of papers (n = 254) did not meet the inclusion criteria, or a neurological disease was investigated. Therefore, 25 full-text articles remained to be assessed for eligibility. Another seven studies had to be excluded because they did not fulfill the inclusion criteria. Finally, 18 studies were included for further analysis.

### Methodological quality

Following “The Cochrane Collaboration`s tool for assessing risk of bias”, all studies showed a high or unclear risk of bias concerning random sequence generation, and blinding of participants and personnel. None of the studies provided information about blinding of outcome assessments. Two studies gave some information about randomization [9,13], 14 studies showed a low risk of incomplete outcome data and 16 a low risk of reporting bias. All studies featured an unclear risk of other bias due to unclear prescription (see Table 1). Moreover, most of the studies had a small sample size (N<30).

**Table 1:** Cochrane risk of bias analysis.

Author, year	Random sequence generation (selection bias)	Allocation concealment (selection bias)	Blinding of participants and personnel (performance bias)	Blinding of outcome assessment (detection bias)	Incomplete outcome data (attrition bias)	Selective reporting (reporting bias)	Other bias
Huber, 2013 [10]	?	?	-	?	+	+	?
Huber, 2011 [20]	?	?	-	?	-	+	?
Imida, 2011 [29]	?	?	-	?	?	?	?
Jaitner, 2010 [18]	?	?	-	?	+	+	?
Kuntze, 2015 [15]	?	?	?	?	+	+	?
Nüesch, 2012 [24]	?	?	?	?	+	+	?
Nurse, 2005 [26]	-	-	-	?	+	+	?
Stirling, 2011 [17]	?	?	?	?	+	+	?
von Tscharner, 2010 [25]	-	-	?	?	+	+	?
von Tscharner, 2010 [14]	?	?	?	?	+	+	?
von Tscharner, 2009 [16]	?	?	?	?	-	-	?
von Tscharner, 2006 [13]	?	+	?	?	+	+	?
von Tscharner, 2004 [19]	?	?	-	?	+	+	?
von Tscharner, 2003 [9]	?	?	?	?	?	+	?
von Tscharner, 2003 [21]	?	+	?	?	+	+	?
Wakeling, 2004 [22]	?	?	-	?	+	+	?
Wakeling, 2002 [23]	?	?	?	?	+	+	?
Wakeling, 2001 [6]	?	?	-	?	+	+	?

+ low risk of bias

- high risk of bias

? unclear risk of bias

### Study characteristics

In 18 studies, EMG activity patterns of the lower extremities were investigated: 13 investigated walking, 4 running and one different walking and running conditions. The summary of wavelet analysis of EMG walking data is depicted in Table 2 and of EMG running and different conditions data in Table 3. In the included studies, three main topics were discussed: The recognition rate, time period characteristics, and motor unit characteristics. These three topics are defined in the following section and summarized in Tables 2 & 3.

**Table 2:** Data extraction: Summary of wavelet analysis of EMG of lower extremity muscles while walking.

Main objective	First author, year	Subject characteristics: Intervention Group (IG); Control Group (CG), sample size (n), gender, group-specification, age	Intervention (EMG)	Outcomes Measures & Variables	Results	Wavelet specification
Recognition rate	von Tscharner, 2010 [14]	IG, n = 16, 9 females, 7 males, osteoarthritis, 53.0yr, range 33–74; CG, n = 15, 9 females, 6 males, healthy, 53.0yr, range 27–65	4 leg muscles; self-selected walking speed; 19–22 successful trials within about 1h	Power; cross-validation rate or recognition rate	Average recognition rate of 92.6%, dimension of d = 4 pattern space; spherical classification recognition rate of 98.6%	CF: 19-542Hz; time normalization-duration of stance phase; convolution in time space at time points spaced by 1/300 of stance phase; time and amplitude normalized MMP; spherical classification; Euclidian vector space; principal component analysis; leave-one-out cross validation
	Kuntze, 2015 [15]	IG, n = 10, females, total knee arthroplasty (19±3 month), 61.9±8.8yr; CG, n = 9, females, healthy, 61.4±7.4yr	7 leg muscles; self-selected walking speed; 10m walkaway; 10 valid trials	30% prior stance and 30% after stance phase; recognition rates for MMP and individual muscles	Recognition rates: VM 68.4%, BF 73.7%; altered and delayed activations in different muscles and different stance phases	CF: 19–395 Hz; 10 non-linearly scaled wavelets; normalized to total power; SVM; leave-one-out cross-validation; iterative thresholding approach; remove low level activation across subjects
	von Tscharner, 2004 [19]	IG, n = 2; 1 male, healthy, 46yr, 1 female, healthy, 28yr	7 leg muscles; 3x9 steps without and 3x9 steps with knee brace; 12 days repetition	EMG intensity; discrimination between no knee brace and knee brace	Significant differences in all muscles; less muscle activity VM and VL with knee brace and delayed activity VL	10 non-linearly scaled wavelets; distance versus angel representation (minimal computational effort)
Time period characteristics	Huber, 2013 [10]	IG, n = 10, females, healthy, 48.0±7yr	5 leg muscles; self-selected walking speed; 10m walkaway	Power; time period: 250ms before to 250ms after heel strike; intra-subject PCA, inter-subject PCA; reflex-cycles; pre-activation	Normalized eigenvalues of intra-subject analysis agreed with inter-subject analysis; first PC-vector consistent between subjects while higher vectors differed	CF: 92-395Hz; 13 non-linearly scaled wavelets; normalized by the integrated power = 1; PCA
	Huber, 2011 [20]	IG, n = 10, females, healthy, 48.0±7yr	QF, ST; self-selected walking speed; 10m walkaway	Power; time period: 250ms before to 250ms after heel strike	Sufficiently sensitive to detect a synchronization of muscle activation while walking in a 40ms rhythm; a lot of jitter in the location of activation peaks	CF: 92-395Hz; 13 non-linearly scaled wavelets
Motor unit characteristics	von Tscharner, 2010 [14]	IG, n = 16, 9 females, 7 males, osteoarthritis, 53.0yr, range 33–74; CG, n = 15, 9 females, 6 males, healthy, 53.0yr, range 27–65	4 leg muscles; self-selected walking speed; 19–22 successful trials within about 1h.	Power; cross-validation rate or recognition rate	Shift of EMG intensity to lower frequencies in the affected leg; time shift—muscular activity occurred earlier in patients	CF: 19-542Hz; time normalization-duration of stance phase; convolution in time space at time points spaced by 1/300 of stance phase; time and amplitude normalized MMP; spherical classification; Euclidian vector space; principal component analysis; leave-one-out cross validation
	von Tscharner,2010 [25]	IG, subjects with osteoarthritis	4 leg muscles; healthy and affected leg	Pattern recognition method	Activity drops to lower frequencies in the affected leg during stance phase; shift in timing–SO activated earlier; 70% correctly assigned	CF: 19-542Hz; Wavelet transform described by von Tscharner, 2000; spherical classification procedure
	Nuesch, 2012 [24]	IG, n = 12, 6 females, 6 males, osteoarthritis, mean (SEM) 56.6yr (3.34); CG, n = 12, 5 females, 7 males, healthy, mean (SEM) 48.4yr (3.12)	7 leg muscles; six walking trials at self- selected speed	Power; homogeneity of muscle activation: entropy of each individual wavelet pattern	Shift toward lower frequencies in the mean wavelet spectrum of TA, intensity lower; SO and PL higher entropy in patients	CF: 19-395Hz; 10 non-linearly scaled wavelets; normalized to total power; wavelet pattern divided into four frequency regions: w1-w3, w4-w5, w6-w8, w9-w10
	Nurse, 2005 [26]	IG, n = 15, 3 females, 12 males, 24.7±2.9yr	7 leg muscles, walking speed 1.5m/s; 30m walkaway; two shoes insert conditions	Total EMG intensity; low and high frequency components; EMG signals were calculated for the first 20%, 20–70% and the final 30% of stance	Textured insert caused reduction of SO and TA energy in low but not in high frequency domain of the entire stance phase	CF: 7–395 Hz; 11 wavelets; discrete time intervals; Gaussian filter; data from each muscle were normalized to each subject`s mean energy for the flat insert condition
	Imada, 2011 [28]	IG, n = 5, males, healthy, 24.5±2.1yr	Gmed and Gmax; 3x5m walking	Identify characteristics of type II fibers during changing direction	High frequency during changing direction indicated much higher than straight walking.	High frequency over 80Hz; EMG—normalized to integrated EMG with 5% interval width.

*Muscles*: QF: Quadriceps femoris, VM: Vastus medialis, VL: Vastus lateralis, BF: Biceps femoris, Gmed: Gluteus medius, Gmax: Gluteus maximus, TA: Tibialis anterior, PL: Peroneus longus, SO: Soleus *Methods*: CF: Center Frequency; PCA: Principal Component Analysis; SVM: Support Vector Machine; MMP: Multi Muscle Activation Pattern

**Table 3:** Data extraction: Summary of wavelet analysis of EMG of lower extremity muscles while running and different conditions.

Main objective	First author, year	Subject characteristics: Intervention Group (IG); Control Group (CG), sample size (n), gender, group-specification, age	Intervention(EMG)	Outcomes: Measures & Variables	Results	Wavelet specification
Recognition rate	von Tscharner, 2003 [9]	IG, n = 81, 41 females, 40 males, healthy runners	5 leg muscles; speed of 4m/s; 5 trials in each of 3 footwear conditions	Introduce a discriminant window showing how the EMG of two groups of subjects can be used to discriminate between them in pattern space; gender differences	More than 95% correct classifications as men or women	11 non-linearly scaled wavelets; normalized to magnitude; MMPs; PCA; intensity patterns represented as vector in pattern space; t-limit = 0.75
	von Tscharner, 2009 [16]	IG, n = 81, 41 females, 40 males, healthy runners	5 leg muscles; speed of 4m/s; 5 trials in each of 3 footwear conditions	Recognition rate; distinguish the footwear condition; averaged intensity patterns of the five data collection trials	High similarity from barefoot and shoed running. Maximal recognition rate of 87% barefoot and shod; average rate: 84%; barefoot and shoe 1: 83%; average rate: 80%; no difference shoe 1 and 2	CF: 19-395Hz; 11 non-linearly scaled wavelets; Cauchy wavelets as a base (symmetry and bell-shaped frequency representation); normalized intensity patterns; PCA displayed as MMP; spherical classification; leave-one-out
	Jaitner, 2010 [18]	IG, n = 8, 5 males, 3 females, healthy athletes, 18.6±2.4yr	8 leg muscles; 5 times 200m; treadmill; at different conditions	Recognition of individual EMG patterns	Recognition rates: pattern to individual: 92.9–100%; different speed and incline: 78.6–88.2%	11 non-linearly scaled wavelets; (von Tscharner, 2000); SVM
	Stirling, 2011 [17]	IG, n = 15, females, healthy, recreational runners, 30.8±7.6yr	4 leg muscles; 1-h treadmill run at 95% of their maximal speed	300ms window around heel strike; discrimination between different effort phases	Average recognition rates: ST 94.0%, TA 89.2%, GCmed 88.3%, VL 84.6%.	CF: 7-542Hz; 13 non-linear wavelets; SVM; n-fold cross validation; penalty parameter C
Time period characteristics	von Tscharner, 2006 [13]	IG, n = 80, 40 females, 40 males, healthy runners	GCmed, TA; speed of 4m/s; 5 trials in each of 3 footwear conditions	Absolute time difference of the activation of the slow or fast groups of muscle fibers	TA: Intensity increased gradually before heel strike, a sharp drop at heel strike. GC Activation started after heel strike.	CF: 7-395Hz; 10 wavelets; normalized by dividing by the Euclidian norm of the vector representing the intensity pattern; PCA; g-spectra
	von Tscharner, 2003 [21]	IG, n = 40, males, healthy runners	TA; speed of 4m/s; 5 trials in each of 3 footwear conditions	EMG changes in time, intensity and frequency shortly before and after heel strike	TA: Pre-heel strike: EMG activity between -100 and -30ms; heel strike: Intensity dropped to almost 0μV	CF: 7-395Hz; 10 non-linearly scaled wavelets; Gauss filter; CF and time-resolutions were calculated with q = 1.45 and r = 1.959
	Wakeling, 2004 [22]	IG, n = 6, healthy, recreational runners, 33.0±3yr	9 leg muscles; speeds: 1.5m/s, 3m/s and 4.5m/s; each block repeated six times (45s each)	Time—frequency space; mean intensity spectrum was calculated for each time-window	Increased running velocity-myoelectric intensity increased for all muscles; time varying shifts in the motor recruitment patterns	CF: 10-524Hz First wavelet: From heel strike 20 equal time-windows; mean intensity for each muscle and subject for the 4.5m/s trial was calculated and used to normalize the spectra for the respective muscles and subjects; PCA
	Wakeling, 2002 [23]	IG, n = 6, 3 females, healthy, recreational runners, 23.3±4.1yr, 3 males, healthy, recreational runners, 26.0±2.5yr	4 leg muscles; two 30min running trials per week for 4 weeks; two shoes	Pre-contact EMG intensity for the 150ms pre-heel strike; mean intensity, total intensity	Significant changes between shoes, subjects and muscles; total EMG intensity 290% for the 150ms pre-heel strike time window; muscle specific	CF: 10-430Hz; low band (25–75 Hz), high band (150-300Hz)
	Wakeling, 2001 [6]	IG, n = 6, 5 females, healthy, proficient runners, 32.3±1.6yr, 1 male, healthy, proficient runner, 29.7yr	4 leg muscles; 30min running at two testing session	Intensity -150ms to heel strike, heel strike to 150ms	Changes in muscle recruitment patterns during sustained sub-maximal running	CF: 11–370 Hz; 9 wavelets; low band (40-60Hz), high frequency band (170-220Hz); Gauss filter; pooled data to determine the general pattern of rates of change
Motor unit characteristics	von Tscharner, 2003 [9]	IG, n = 81, 41 females, 40 males, healthy runners	5 leg muscles; speed of 4m/s; 5 trials in each of 3 footwear conditions	Analyze the gender differences in five muscles of the limb of male and female runners	Women: GCmed, HS: Higher low frequency activity; TA: Larger intensities and higher frequencies (100-150Hz); RF: Lower low frequency and higher high frequency components during the first 40ms after heel strike; VM: -25 to -100ms less high frequency components	11 non-linearly scaled wavelets; normalized to magnitude; MMPs; PCA; intensity patterns represented as vector in pattern space; t-limit = 0.75
	von Tscharner, 2006 [13]	IG, n = 80, 40 females, 40 males, healthy runners	GCmed, TA; speed of 4m/s; 5 trials in each of 3 footwear conditions	Absolute time difference of the activation of the slow or fast groups of muscle fibers	TA: Activated by the fast fibers in the pre-heel strike period, the slow fibers controlled the early part of stance phase. GC: Activation started after heel strike and increased the frequency from low to high	CF: 7–395 Hz; 10 wavelets; normalized by dividing by the Euclidian norm of the vector representing the intensity pattern; PCA; g-spectra
	von Tscharner, 2003 [21]	IG, n = 40, males, healthy runners	TA; speed of 4m/s; 5 trials in each of 3 footwear conditions	EMG changes in time, intensity and frequency shortly before and after heel-strike	TA: Pre-heel strike: Minimal intensities for wavelet 1 and 2, substantial intensities for wavelets 3–9; heel strike: Intensity dropped to almost 0μV; after heel strike: Activated around wavelet 4	CF: 7-395Hz; 10 non-linearly scaled wavelets; Gauss filter; CF and time-resolutions were calculated with q = 1.45 and r = 1.959
	Wakeling, 2004 [22]	IG, n = 6, healthy, recreational runners, 33.0±3yr	9 leg muscles; speeds: 1.5m/s, 3m/s and 4.5m/s; each block repeated six times (45s each)	Time—frequency space; mean intensity spectrum was calculated for each time-window	Increased running velocity-myoelectric intensity increased for all muscles; different types of motor unit are recruited in a task-dependent fashion during locomotion	CF: 10–524 Hz First wavelet: From heel strike 20 equal time-windows; mean intensity for each muscle and subject for the 4.5 m/s trial was calculated and used to normalize the spectra for the respective muscles and subjects; PCA
	Wakeling, 2002 [23]	IG, n = 6, 3 females, healthy, recreational runners, 23.3±4.1yr, 3 males, healthy, recreational runners, 26.0±2.5yr	4 leg muscles; two 30min running trials per week for 4 weeks; two shoes	Pre-contact EMG intensity for the 150ms pre-heel strike; mean intensity, total intensity	Significant changes between shoes, subjects and muscles; greatest changes (%) in EMG intensity between 11-75Hz and 192-301Hz	CF: 10-430Hz; low band (25-75Hz), high band (150-300Hz)
	Wakeling, 2001 [6]	IG, n = 6, 5 females, healthy, proficient runners, 32.3±1.6yr, 1 male, healthy, proficient runner, 29.7yr	4 leg muscles; 30min running at two testing session	Intensity -150ms to heel strike, heel strike to 150ms	Pooled data-decrease in the rate of change wavelets 1–3 and an increase in the rate of change for wavelet domains 6–8. The low-frequency components decreased in intensity, the high-frequency components increased in intensity during the 30min running trials	CF: 11-370Hz; 9 wavelets; low band (40-60Hz), high frequency band (170-220Hz); Gauss filter; pooled data to determine the general pattern of rates of change

*Muscles*: GCmed: Gastrocnemius medialis, HS: Hamstrings, ST: Semitendinosus TA: Tibialis anterior, RF: Rectus femoris, VM: Vastus medialis, VL: Vastus lateralis *Methods*: CF: Center Frequency; PCA: Principal Component Analysis; SVM: Support Vector Machine; MMP: Multi Muscle activation Pattern

### Recognition rate

The recognition rate is the capability of a method to correctly assign participants to a specific group [14,15]. Different classification procedures were applied to find the boundaries between two groups under investigation and to improve the accuracy of the method. Intensity patterns which reflect how strong a signal is at time *t*, are sometimes very difficult to assign to a certain condition by visual inspection because the signals can appear inconsistent [1,16]. However, differences between the measured conditions must be reflected in the signal. The combination of wavelet analysis with methods from multivariate statistics and the field of pattern recognition, like principal component analysis (PCA) and support vector machines (SVM) [16], allows to detect subtle differences in muscle activation patterns. PCA is a statistical method to identify components and order them according to their strength to explain the variance in given datasets. It allows reducing the dimensionality in large datasets and hence it also allows uncovering the most important factors. The term “support vector machine” is inspired from their application in machine learning applications, for example face or speech detection by computers. SVMs are suitable for allocating a given set of data to one of two classes, for example to a patient or a control group. Wavelet analysis in conjunction with subsequent PCA to find principal patterns in EMG intensities, was used to discriminate between healthy people and patients with ankle osteoarthritis while walking [14], between barefoot runners and shod runners [16] and males from females [9]. The application of wavelet analysis together with SVMs was used to find the boundaries to discriminate patients with total knee arthroplasty from healthy people while walking [15] or to correctly assign the EMG patterns of different running tasks to each subject [17,18]. Eight studies computed the recognition rate [9,14–19].

### Recognition rate distinguishing between different conditions

The recognition rate discriminated between different running, walking or footwear conditions measured with different muscles of the leg [9,15,17–19]. The maximum recognition rate was 78 to 94.4% and varied between different muscles and methods. Jaitner et al. (2010) [18] tested the recognition of individual EMG patterns of leg muscles with different walking and running conditions with a resulting rate of 92.9 to 100%. Moreover, Kuntze et al. (2015) [15] discriminated total knee arthroplasty patients from healthy people with a rate of 68.4–73.7% while walking. Von Tscharner (2003) [9] determined more than 95% correct gender classification as male or female runners. The recognition method was able to extract gender specific differences in timing, intensity and frequency distribution of electromyographic signals.

### Time period characteristics

The behavior of EMG intensity and frequency over time was analyzed. The non-stationary wavelet approach was used for the evaluation of muscle timing, time shifts and early or delayed muscle activation [6,10,13,15,16,20–23]. Originally, wavelet analyses have used a mother wavelet which waslinearly scaled. However, this method did not represent the physiological response time of the muscles in the higher frequencies [1]. In contrast, the time resolution of non-linearly scaled wavelets described by von Tscharner et al. (2000) [1] represent a more physiological condition. Differences were revealed between the activation patterns of walking versus running.

### Time period characteristics of the lower extremity muscles while walking

In healthy subjects walking presented a pre-activation peak 50–100 ms before heel-strike [10], followed with a sharp decline as a response to the reflex attenuation. This pre-activation was highly subject-specific. For the reflex response with a reaction time to maximum activity between 30–50 ms after heel-strike, similar muscle activation patterns were found. Thus, the reflex response was less subject-specific than the pre-activation [10]. Patients with total knee arthroplasty presented with a delayed peak activity of vastus medialis and biceps femoris muscle in the early stance phase of walking but with a prolonged activation in mid stance of vastus medialis, vastus lateralis, and biceps femoris [15]. The control strategy of a joint while walking, requires a dynamic interplay between muscles, the central nervous system and external factors [20]. According to Huber et al. (2011) [20], neuromuscular control mechanisms showed a precise pacing to control the individual walking pattern. This pacing of neuromuscular activity while walking, seemed to be controlled as a programmed function at 40 ms intervals, independent from subject and muscle. Nonetheless, the neuromuscular rhythm was less precisely controlled while walking than while running, reflected as many variations in timing and intensity of muscle activation [20].

### Time period characteristics of the lower extremity muscles while running

Electromyographic signals have been found to be very subject-specific [16,23]. Therefore, EMGs of different muscles have been arranged into Multi-Muscle activation Patterns (MMPs). To form a MMP the intensity patterns of individual muscles have been stacked one above the other. Thus, the MMPs represented the intensity of the electromyographic signals of several muscles simultaneously. These resulting MMPs demonstrated a pattern with high similarity of muscle activation while running [6]. The EMG activation of the gastrocnemius presented between 0 (heel-strike) and 200 ms after heel-strike and of the tibialis anterior between -100 and 150 ms [13]. Altered shoe conditions influenced the timing of tibialis anterior minimally in pre-heel-strike anticipation but delayed for the post-heel-strike reflex. There was a shift for the maximum EMG intensity from 25 ms after heel-strike in the barefoot condition to 60 ms in the shoed conditions [21]. Between different running speeds differences have been presented in motor unit recruitment patterns. Overall, the higher the running speed the higher the intensity for all muscles tested [22].

### Motor unit characteristics

Recruitment patterns of slow type I to fast type II fibers have been investigated by means of the intensity levels at different wavelet frequency bands [13]. Intensities below 100Hz simultaneously reflected the activity of slow and fast muscle fibers; above 100Hz fast muscle fibers dominated [13].

### Motor unit characteristics of the lower extremity muscles while walking

Down shifted EMG spectra of tibialis anterior toward lower frequencies have been observed in patients with ankle osteoarthritis. The wavelet patterns of patients showed a trend towards less intensity production of tibialis anterior between 170 and 271Hz but more intensity between 19 and 62Hz [24]. Atrophic muscles have been demonstrated to present relatively less intensity in high frequency bands and more in the lower frequencies [14,24,25]. Textured insoles caused a reduction in the intensity of the electromyographic signal of the soleus and tibialis anterior in the low (38-62Hz) but not in the high frequency (170-271Hz) domains and this continued throughout the entire stance phase [26].

### Motor unit characteristics of the lower extremity muscles while running

Motor unit characteristics while running have been mainly investigated by Wakeling et al. and von Tscharner et al. [6,13,21–23]. They showed that the tibialis anterior was activated by the fast fibers (60-270Hz) in the pre-heel-strike period followed by a drop in intensity at heel-strike. The slow fibers (10-90Hz) controlled the early part of the stance phase. The two spectrally very different time periods were only 15 ms apart [13,21]. Wearing shoes resulted in an increase of the pre-heel-strike activity of the tibialis anterior and in a decrease of post-heel-strike activity of its slow muscle fibers [26]. Changes in shoe hardness resulted in changes for the tibialis anterior in the intensity of the EMG, both positively and negatively at high (192-301Hz) and low frequency (11-75Hz) bands in a subject-specific manner. These changes were consistent with changes in the recruitment patterns between fast and slow motor units [23]. The activation of the gastrocnemius started with low frequency components after heel-strike and shifted gradually to higher frequency components. The highest frequencies were recorded 110 ms after heel-strike. However, 160 ms after heel-strike, predominant activities of slow muscle fibers were recorded [13]. The mean frequency of hamstrings dropped from 60Hz at the pre-heel-strike maximum to 40Hz at heel-strike. At 120 ms, a slightly higher maximum was reached compared to the pre-heel-strike maximum. The mean frequency of the quadriceps femoris had already increased during pre-heel-strike [9]. During 30 minute running trials, the low frequency components decreased and the high frequency components increased in intensity [6,22]. Regarding gender differences, women activated more high frequency components of tibialis anterior, between -300 and -70 ms, but less between -70 ms and heel-strike than men. Women tended to activate muscle fibers in the lower frequency components [9].

## Discussion

The aim of this systematic review was to demonstrate how wavelet analyses applied to EMG of lower extremity muscles have successfully differentiated between different walking and running conditions, and between subjects with and without certain orthopedic conditions.

### Risk of bias

Since none of the studies reviewed were designed as randomized control trials, the issues of randomization and blinding are not relevant. The study designs of the included studies were appropriate regarding the research questions.

### Recognition rate

Non-linearly scaled wavelets, as originally described by von Tscharner (2000) [1] have been shown to discriminate not only between walking and running, but can also detect difference in muscle activity during ambulation in patients with different orthopedic conditions [9,14–19]. Therefore, these techniques might be clinically useful to detect locomotor dysfunction, as well as help design therapeutic interventions (such as orthotic devices, insoles, etc.) to treat or prevent musculoskeletal problems of the lower extremities.

### Time period

The use of wavelets for processing non-stationary EMG has been shown to be suitable for evaluating muscle timing, time shifts and early or delayed muscle activation [6,10,13,15,16,20–23]. The major sources of the variability in walking [10,14,15,20] and running [6,13,16,22,23] could be evaluated with wavelets. It was shown that the heel-strike event is an important source of this variability as each walking or running step requires a special adaption to the given condition [10]. However, the neuro-muscular control system reacts at each heel-strike with muscular preparation (tuning) and adaptation. Wavelet analyses made it possible to analyze this muscular preparation a few milliseconds before heel-strike and the muscular adaption after heel-strike considering time, intensity and frequency content. The neuromuscular control mechanisms showed a precise pacing at 40 ms intervals to control the individual walking pattern [20]. This periodic, cortical rhythm (Piper rhythm) seemed to represent the fine tuning needed for controlled movements [20]. Maurer et al. (2013) found that the Piper rhythm varied with running speed [27]. It was supposed that the frequency adapted to exterior condition and modulated the muscle activity between on and offset. The mean frequency of the Piper rhythm increased from 28 to 36 Hz as running speed decreased from 4.9 to 1.3 m/s. The authors assumed that very slow running speeds are not natural and therefore, require more efficient control [27]. However, the neuromuscular rhythm was less precisely controlled while walking than while running, reflected as many variations in timing and intensity of muscle activation [20,27]. These findings are consistent with the assumption that running is more tightly controlled and less variable [27].

This knowledge of muscular preparation and adaption to variable heel-strike events, as well as the interplay of the muscles are useful regarding training protocols in sports and in rehabilitation [25]. It allows athletes and therapists to monitor the training of specific muscles, to optimize the movements and to observe the training progress [25]. Muscles of patients with ankle osteoarthritis or total knee arthroplasty were activated earlier and for longer periods than in healthy people [14,15]. It was assumed that the central nervous system would have to activate the selected motor units of these patients earlier to obtain the required force at heel-strike because of the lower conduction velocity or the longer electromechanical delay of the selected fibers [14,28]. Furthermore, as significant differences were found in the lower extremity muscles [22], the influence of different locomotion speeds and gait variations should be considered in further studies.

### Motor unit characteristics

Fast type II muscle fibers generate higher rates of force development than slow type I fibers, and are more likely to allow a fast release compared to slow fibers [21]. During the impact of heel-strike, a fast development of force in the tibialis anterior muscle is needed to stabilize the joints. After heel-strike a fast relaxation is important as well to release the forefoot. This explains that the highest frequencies have been found just before heel-strike [21]. However, task specific recruitment of the slower, fatigue resistant muscle fibers have been preferentially recruited while walking [26]. It is supposed that fast and slow twitched fibers are recruited to match their contractile properties to the mechanical requirements for motion, and these requirements change between gait mode, speeds and within stride [22]. Changes in shoe materials resulted in changes in the intensity ratio of the EMG at high and low frequency bands [23]. Wakeling et al. (2002) interpreted the findings as a response to altered impact forces. The spectral changes of the tibialis anterior muscle towards lower frequencies in patients with ankle osteoarthritis, has been associated with a selective atrophy of type II muscle fibers. Changes in muscle fiber recruitment while walking or running may provide information for prevention and treatment of runners. However, to verify this assumption, histological studies (not only in animals as used as theoretical basis in the studies) are needed [14,24]. A strong and critical debate has been held on this topic. Farina (2008) criticized that von Tscharner et al. did not discuss and consider the electrophysiological properties associated with different fiber types [29]. Furthermore, it was mentioned that the EMG power spectrum can be influenced by many different factors and therefore, the influence of selected physiological variables will probably be small. Farina (2008) noted that the use of spectral analysis of EMG to measure motor unit recruitment or the properties of muscle fiber types has not been validated [29].

### Strengths and limitations

To the best of our knowledge, this is the first systematic review that summarizes the use of wavelet analyses of electromyographic recordings from lower extremity muscles during walking, running, and certain orthopedic conditions. The influence of the interdisciplinary knowledge of the team including physiotherapists, sports and movement scientists, biomechanics and engineers, all experienced in signal analysis is another strength of the review.

However, the study has the limitation that in a couple of studies low numbers of participants were included. Furthermore, almost all reviewed articles applied the non-linearly scaled wavelet method developed by von Tscharner (2000) [1]. However, other kinds of wavelets with other time/frequency resolution might resolve other aspects of the interplay of muscles with different dynamic motor tasks. This aspect should be considered in further studies.

## Conclusion

Wavelets derived from the lower extremity muscles’ EMG reflect signal components related to activities of small and large alpha-motoneurons and also muscle timing and intensity characteristics. The simultaneous information of time, frequency and intensitiy behavior is of high clinical relevance because it offers valuable information about pre- and reflex activation behavior on different walking and running speeds as well as spectral changes towards high or low frequencies at every time point of the gait cycle. Furthermore, the required information of motor unit recruitment behavior in the pre- and post-heel-strike phase as well as at heel-strike could be extracted, which sheds light on specific differences of muscle activation patterns. This knowledge allows for optimizing training and rehabilitation protocols. The optimized accuracy of discriminating between healthy people and people with orthopedic conditions wavelets provide a useful diagnostic tool to analyze group differences or pre-/post study designs.

## Supporting information

S1 Fig SR_PRISMA 2009 checklist. (DOC)

## Abbreviations

EMG: Electromyogram
FT: Fourier Transform
MMPs: Multi-Muscle activation patterns
PCA: Principal Component Analysis
PFM: Pelvic Floor Muscles
RCT: Randomized controlled trial
SVM: Support Vector Machine
